# Diffusion tensor imaging tractography reveals altered fornix in all diagnostic subtypes of multiple sclerosis

**DOI:** 10.1002/brb3.1514

**Published:** 2019-12-19

**Authors:** Diana Valdés Cabrera, Robert Stobbe, Penelope Smyth, Fabrizio Giuliani, Derek Emery, Christian Beaulieu

**Affiliations:** ^1^ Department of Biomedical Engineering University of Alberta Edmonton AB Canada; ^2^ Department of Medicine University of Alberta Edmonton AB Canada; ^3^ Department of Radiology University of Alberta Edmonton AB Canada

**Keywords:** diffusion tensor imaging, fornix, limbic system, mri, multiple sclerosis, tractography

## Abstract

**Introduction:**

Diffusion tensor imaging (DTI) has shown abnormalities of the fornix and other limbic white matter tracts in multiple sclerosis (MS), mainly focusing on relapsing‐remitting MS.

**Methods:**

The goal here was to evaluate the fornix, cingulum, and uncinate fasciculus with DTI tractography at 1.7 mm isotropic resolution in three MS subgroups (11 relapsing‐remitting (RRMS), nine secondary progressive (SPMS), eight primary progressive (PPMS)) versus 11 controls, and assess correlations with cognitive and clinical scores.

**Results:**

The MS group overall showed extensive diffusion abnormalities of the fornix with less volume, lower fractional anisotropy (FA), and higher mean and radial diffusivities, which were similarly affected in all three MS subgroups. The uncinate fasciculus had lower FA only in the secondary progressive subgroup, and the cingulum had no DTI differences in any MS subgroup. The FA and/or volumes of these tracts correlated negatively with larger total lesion volume. The only DTI‐cognitive correlation was lower right cingulum FA and greater depression over the entire MS cohort.

**Conclusions:**

Diffusion tractography identified abnormalities in the fornix that appears to be affected early and consistently across all three primary MS phenotypes of RRMS, SPMS, and PPMS regardless of Expanded Disability Status Scale, time since diagnosis, or cognitive scores.

## Graphical abstract

Multiple sclerosis (MS) often results in cognitive dysfunction, fatigue, and depression, which may have a microstructural basis in limbic white matter tract degradation. High‐resolution diffusion tensor imaging (DTI) tractography at 4.7T assessed the fornix, cingulum, and uncinate fasciculus tracts and their links to clinical/cognitive scores in three MS diagnostic subgroups. The entire MS cohort showed diffusion abnormalities of the fornix with less volume, lower fractional anisotropy (FA), and higher mean and radial diffusivities, which were similarly affected in all three MS subgroups, and FA and/or volumes of these tracts correlated negatively with larger total lesion volume regardless of Expanded Disability Status Scale, time since diagnosis, or cognitive scores.

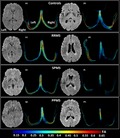

## 1 INTRODUCTION

Inflammation, demyelination, and axonal degeneration of white matter (WM) pathways are hallmarks of multiple sclerosis (MS; Bjartmar, Wujek, & Trapp, 2003). The progressive deterioration of specific WM tracts may cause disconnections between cortical/subcortical regions that are associated with particular cognitive impairments (Mesaros et al., 2012). Diffusion tensor imaging (DTI) is well suited to the virtual identification of WM pathways and can yield quantitative metrics that are sensitive to microstructural damage of WM, not just limited to the clinical‐MRI visible lesions in MS. A whole‐brain meta‐analysis of the WM “skeleton” using tract‐based spatial statistics (TBSS) has shown lower fractional anisotropy (FA) in the corpus callosum, which has been a major focus of prior work due to its preferential location for MS lesions and its large size, but also in smaller tracts such as the fornix and cingulum where lower FA has been associated with greater physical disability and impaired cognition (Welton, Kent, Constantinescu, Auer, & Dineen, 2015). Separate whole‐brain analyses of mostly relapsing‐remitting MS (RRMS) participants have reported lower FA throughout much of the WM with FA in some specific WM regions (e.g., again including cingulum and fornix) correlating to deficits such as processing speed and working memory (Dineen et al., 2009; Kern et al., 2012; Roosendaal et al., 2009; Yu et al., 2012).

Tracts of the limbic system, like the fornix, play crucial roles in aspects of cognition, memory, behavior, and reasoning (Catani & de Schotten, 2012), and they have been implicated in the above whole‐brain analyses of MS. Given this relevance, limbic system tracts have been the focus in MS diffusion MRI studies assessed with either region of interest/atlas (Dineen, Bradshaw, Constantinescu, & Auer, 2012; Koenig et al., 2013; Mesaros et al., 2012; Pardini, Bergamino, et al., 2014; Van Geest et al., 2018) or tractography (Fink et al., 2010; Kern et al., 2015; Keser et al., 2017; Louapre et al., 2016; Syc et al., 2013), both targeted analyses that can have distinct advantages over whole‐brain voxel‐based methods (e.g., far fewer multiple comparisons and do not need intersubject registration for native space tractography). The majority of these DTI studies have been in patients with RRMS, but FA abnormalities in the limbic WM tracts, including the fornix, have also been demonstrated in primary progressive (PPMS; Bodini et al., 2009) and secondary progressive MS (SPMS) (Meijer et al., 2016). One such voxel‐wise whole‐brain DTI study showed that PPMS had widespread diffusion abnormalities in WM and that SPMS had more extensive WM diffusion changes than either RRMS or PPMS (Preziosa, Rocca, & Caputo, 2011).

Three key limitations can be identified from these limbic white matter DTI studies of MS. First, with the exception of the latter three studies above (Bodini et al., 2009; Meijer et al., 2016; Preziosa et al., 2011), analyses were based on mainly RRMS participants or analyses that treated all MS patients as a single cohort. Second, the fornix is a very small tract (~3, 3.5, and 6.5 mm mean cross‐sectional diameters in the posterior and anterior columns and body, respectively, even in healthy participants; Pascalau, Stănilă, Sfrângeu, & Szabo, 2018) that passes through cerebrospinal fluid (CSF) and hence is not ideal for analysis when DTI data are acquired at typical low spatial resolutions (e.g., >2 mm isotropic, voxel size >8 mm^3^). Third, the typically used TBSS voxel‐wise analysis of the WM has been shown to have particular difficulty with the FA skeleton of the fornix (Bach et al., 2014).

The purpose of this study was to (a) use DTI deterministic tractography at high field strength (4.7T) and spatial resolution of 1.7 mm isotropic to investigate whether microstructural damage to specifically the small limbic tracts (fornix, cingulum, and uncinate fasciculus) is similar in all three diagnostic subgroups of MS (RRMS, SPMS, and PPMS), and (b) correlate the DTI‐derived tract diffusion metrics and volumes of the entire MS cohort with both lesion numbers/volumes and cognitive impairments (visuospatial memory speed, depression, and fatigue), disability (EDSS), and time since diagnosis.

## 2 MATERIALS AND METHODS

### 2.1 Participants

This study was approved by the University of Alberta Human Research Ethics Board. All 39 participants gave written informed consent including 28 diagnosed with MS (11 RRMS, nine SPMS, and eight PPMS) that were recruited from the University of Alberta MS Clinic, as well as 11 typical controls with no history of neurological/psychiatric disease or brain injury that covered the same age range and had similar sex distribution. MS participants were not targeted for their subtype, but a goal was to have similar proportions from RRMS, SPMS, and PPMS with a target MS sample of 30 total (28 actual) given other constraints. There were no patients with relapses or taking steroids at the time of the MRI scan. However, there was no available information regarding whether they had recent prior relapses or not. Therefore, none of the MS participants were excluded from the study. The SPMS and PPMS participants were not receiving medications or treatments specific for MS at the time of the MRI study, except one SPMS participant on IV infused natalizumab. Three RRMS participants were not taking any medications, but the other 8 RRMS were on different disease‐modifying therapies (dimethyl fumarate, glatiramer acetate, fingolimod, and natalizumab). Three participants (RRMS, SPMS, and PPMS) were on antidepressant medications (escitalopram, duloxetine, and bupropion). The demographic and clinical data are summarized in Table 1.

**Table 1:** Demographics for controls and the MS subgroups with range and mean ± *SD* where appropriate (*p ≤ .05 for differences between MS subgroups)

	Controls (*n* = 11)	MS Cohort (*n* = 28)	RRMS (*n* = 11)	SPMS (*n* = 9)	PPMS (*n* = 8)
Sex (M/F)	4/7	10/18	2/9	2/7	6/2*
Age (years)	21–75	21–66	21–58	45–66	41–65
	44 ± 16	48 ± 12	40 ± 12*	55 ± 7	54 ± 7
Time since diagnosis (years)	–	1–34	1–28	2–34	3–17
		13 ± 10	9 ± 8	22 ± 9*	8 ± 6
Expanded Disability Status Scale, EDSS a	–	1.5–8.5	1.5–6	4–8.5	4–7
		4.8 ± 1.7	3.5 ± 1.5*	5.6 ± 1.5	5.8 ± 1
Brief Visuospatial Memory Test‐Revised, BVMT‐R b	–	5–34	10–34	11–34	5–28
		21 ± 9	23 ± 8	21 ± 9	17 ± 9
Symbol Digit Modalities Test, SDMT	–	23–89	36–71	34–89	23–62
		52 ± 14	52 ± 9	57 ± 17	46 ± 13
Paced Auditory Serial Addition Test, PASAT	–	19–59	37–54	19–59	30–45
		42 ± 9	44 ± 7*	43 ± 12	36 ± 6*
Fatigue Severity Scale, FSS	–	1.1–7	2.9–7	1.1–6.9	3.5–7
		5.0 ± 1.5	5.0 ± 1.3	4.7 ± 1.7	5.4 ± 1.3
Modified Fatigue Impact Scale, MFIS	–	2–73	2–73	21–73	34–64
		42 ± 19	38 ± 23	39 ± 19	50 ± 11
Beck Depression Inventory‐II, BDI‐II	–	0–31	0–31	0–29	5–21
		13 ± 9	12 ± 10	14 ± 10	13 ± 7
Timed 25‐Foot Walk, T25‐FW (s)	–	3.6–20.6	3.6–12.0	5.3–19.5	5.8–20.6
		8.5 ± 4.8	6.5 ± 2.4	10.8 ± 6.0	9.7 ± 6.3
9‐Hole Peg Test, 9‐HPT (s)	–	18.7–65.2	18.7–39.8	19.2–54.1	20.7–65.2
		29.4 ± 10.8	24.9 ± 5.6	28.9 ± 11.0	36.2 ± 14.0
Total lesion volume (cm^3^)	–	0.19–37.2	0.23–8.82	0.19–27.9	0.83–37.2
		7.1 ± 8.8	3.0 ± 3.1	9.3 ± 9.1	10.2 ± 12
Total lesion number (range, mean ± SD)	–	3–32	4–24	3–32	7–24
		15.9 ± 7.4	13.1 ± 7.3	18.3 ± 8.4	16.9 ± 5

a EDSS was not acquired in two PPMS participants. BVMT‐R, PASAT, T25‐FW, and 9‐HPT were not acquired in a SPMS participant. PASAT, T25‐FW, and FSS were not acquired in a PPMS participant. Two SPMS and three PPMS participants were not able to complete the T25‐FW.

b Only the Total Recall score of the BVMT‐R was reported.

### 2.2 Cognitive assessment

Cognitive and clinical tests were performed by experienced MS research nurses from the University of Alberta MS Clinic in order to characterize the MS population and to correlate with quantitative MRI metrics. The tests consisted of Kurtzke Expanded Disability Status Scale (EDSS) for overall disability in MS, Brief Visual Memory Test‐Revised (BVMT‐R) for visuospatial learning and memory (Total Recall score only), Symbol Digit Modalities Test (SDMT) for visual scanning/tracking memory and decision making, Paced Auditory Serial Addition Test (PASAT) for auditory information processing speed, Fatigue Severity Scale (FSS) for degree of fatigue, Modified Fatigue Impact Scale (MFIS) for effects of fatigue in terms of physical, cognitive, and psychosocial functioning, Beck Depression Inventory‐II (BDI‐II) for depression, Timed 25‐Foot Walk (T25‐FW) for mobility and leg function, and 9‐Hole Peg Test (9‐HPT) for upper extremity function (dominant/nondominant hand raw scores averaged).

### 2.3 MRI protocol and analysis

MRI was acquired on a Varian Inova 4.7 T MRI with a birdcage transmit and 4‐channel receive array coil. Fast spin‐echo fluid‐attenuated inversion recovery (FSE FLAIR) was acquired with FOV 256 × 192 mm^2^, 1 × 1 mm^2^ in‐plane resolution, 38 4‐mm slices (no gap), TR = 34 s, TE = 204 ms, TI = 3,000 ms, and scan time 3:24 min. DTI was acquired with single‐shot spin‐echo echo‐planar imaging (EPI), GRAPPA R = 2, 80 1.7‐mm slices (no gap) and coverage of 136 mm, FOV 218 × 238 mm^2^, matrix 128 × 140, 1.7 × 1.7 × 1.7 = 4.9 mm^3^ voxel resolution, zero‐filled to 0.85 × 0.85 mm^2^ in‐plane, TR = 9,500 ms, TE = 54 ms, 30 directions, b = 1,000 s/mm^2^, 5 b_0,_ and scan time 6:13 min.

MS lesions were segmented on FLAIR by a probabilistic lesion prediction algorithm in the Lesion Segmentation Tool (LST) MATLAB toolbox (v2.0.15) for SPM (Schmidt et al., 2012) to yield total lesion number (TLN) and volume (TLV), and all the lesion map results were visually checked. DTI was corrected for subject motion and eddy current distortions with the Artefact correction in diffusion MRI (ACID) toolbox (SPM12), and none of the datasets were discarded for excessive movement (all were <2° rotations and 2 mm translations). The ACID rotation and translation corrections for the axial, sagittal, and coronal planes for each subgroup were not significantly different between the subgroups (data not shown).

The brain was extracted with BET (Brain Extraction Tool) in FSL. Gibbs ringing, EPI distortion corrections, and tensor fitting using RESTORE were performed in ExploreDTI (v4.8.6) (Leemans, Jeurissen, Sijbers, & Jones, 2009). Deterministic tractography was performed with an FA threshold of 0.2, a turning angle of 30°, step size of 1 mm, and minimum fiber length of 10 mm. These tracking parameters, primarily FA and angle thresholds, range from 0.13–0.3 and 30°–70°, respectively, in previous healthy and MS cohorts (Concha, Gross, & Beaulieu, 2005; Malykhin, Concha, Seres, Beaulieu, & Coupland, 2008; Fink et al., 2010; Syc et al., 2013; Kern et al., 2012, 2015; Keser et al., 2017).

Tractography analysis was performed by the first author (DVC), who was blinded to group classification, based on published protocols for the limbic system tracts (Concha et al., 2005; Malykhin et al., 2008) which reported excellent interrater/intrarater intraclass correlations (ICC ≥ 0.8) and percent coefficients of variation (CV ≤ 2.5%) for FA and MD. Fornix: “SEED” ROIs were placed on an axial slice around the fornix column above the anterior commissure and in two coronal slices, one between the crus and fimbria, and one in the fornix body. These ROIs were placed on three distinct portions for the left and right hemispheres separately at first, and then, the two fornices were concatenated into one. “NOT” ROIs were placed on axial and coronal slices above, anterior and posterior to the fornix to avoid spurious fibers when necessary. Cingulum: “SEED” ROIs were placed in three coronal slices at the axial location of the genu, mid‐body, and splenium of the corpus callosum in order to track the superior/dorsal portion of the cingulum, including short u‐fibers, and a “NOT” ROI in a midbrain sagittal slice to avoid fibers of corpus callosum. The ventral portion projecting to the entorhinal cortex was not tracked. Uncinate fasciculus (UF): Two “AND” ROIs were placed in the coronal slice where the frontal and temporal lobes are still separated and one ROI in an axial slice in the anterior temporal lobe, just lateral to the amygdala. “NOT” ROIs were placed posterior to the UF. The left and right cingulum and UF were reconstructed separately. Volume, FA, mean (MD), axial (AD), and radial (RD) diffusivities were calculated in ExploreDTI over the entire tracts.

Along‐the‐tract analysis also was performed in ExploreDTI bilaterally in the body of the fornix, which was the fornix tract area consistently identified across the whole MS cohort. The body was segmented by using two “AND” ROIs placed on the columns and the commissural area of the fornix just at the start of the left and right crus to guarantee streamlines connecting both ROIs. FA was calculated along 35 tract location points from anterior to posterior.

### 2.4 Statistical analysis

Statistical analysis was performed using Minitab 19. Multivariate normality was confirmed for all diffusion and volume parameters for each of the three WM tracts under study with a matrix of bivariate scatter plots (data not shown), and outliers were visually inspected by plotting Q–Q plots of the Mahalanobis distances with chi‐square test distribution and five degrees of freedom (volume, FA, MD, AD, and RD). Demographics for controls and MS were compared via two‐sample *t* test for the whole MS cohort, while MS subgroups were compared with ANOVA for numerical data and chi‐square *t* test for categorical data (*p* ≤ .05). FLAIR‐derived total lesion number and volume, and DTI‐derived diffusion parameters (average of right and left) and volumes (total right and left) of the three WM tracts were compared between the MS subgroups and healthy controls with a one‐way ANOVA and a post hoc Tukey's HSD test to control the family‐wise error rate and to check for differences among diagnostic subgroups. The significance levels were corrected for multiple comparisons by using the Benjamini–Hochberg false discovery rate (FDR‐BH) method in MATLAB R2017a (*p* ≤ .011). The FA values along 35 tract points in the left and right fornix body were compared with a general linear model (two‐way ANOVA) to assess along‐the‐tract FA differences per group (FDR‐BH‐corrected *p* ≤ .032 for the left fornix body and *p* ≤ .029 for the right fornix body).

For the combined MS group (*n* = 28), partial Pearson correlation coefficients corrected for age and sex assessed the relationship between FA and volume for each tract (right and left kept separate), total lesion number and volume, cognitive/clinical scores related with the limbic system functions (EDSS, BVMT‐R, SDMT, MFIS, and BDI‐II), and time since diagnosis. Correlation coefficients were controlled for multiple comparisons with the FDR‐BH method (*p* ≤ .0086).

## 3 RESULTS

### 3.1 MS subgroup characteristics, lesion numbers, and volumetric load

**Figure 1:**
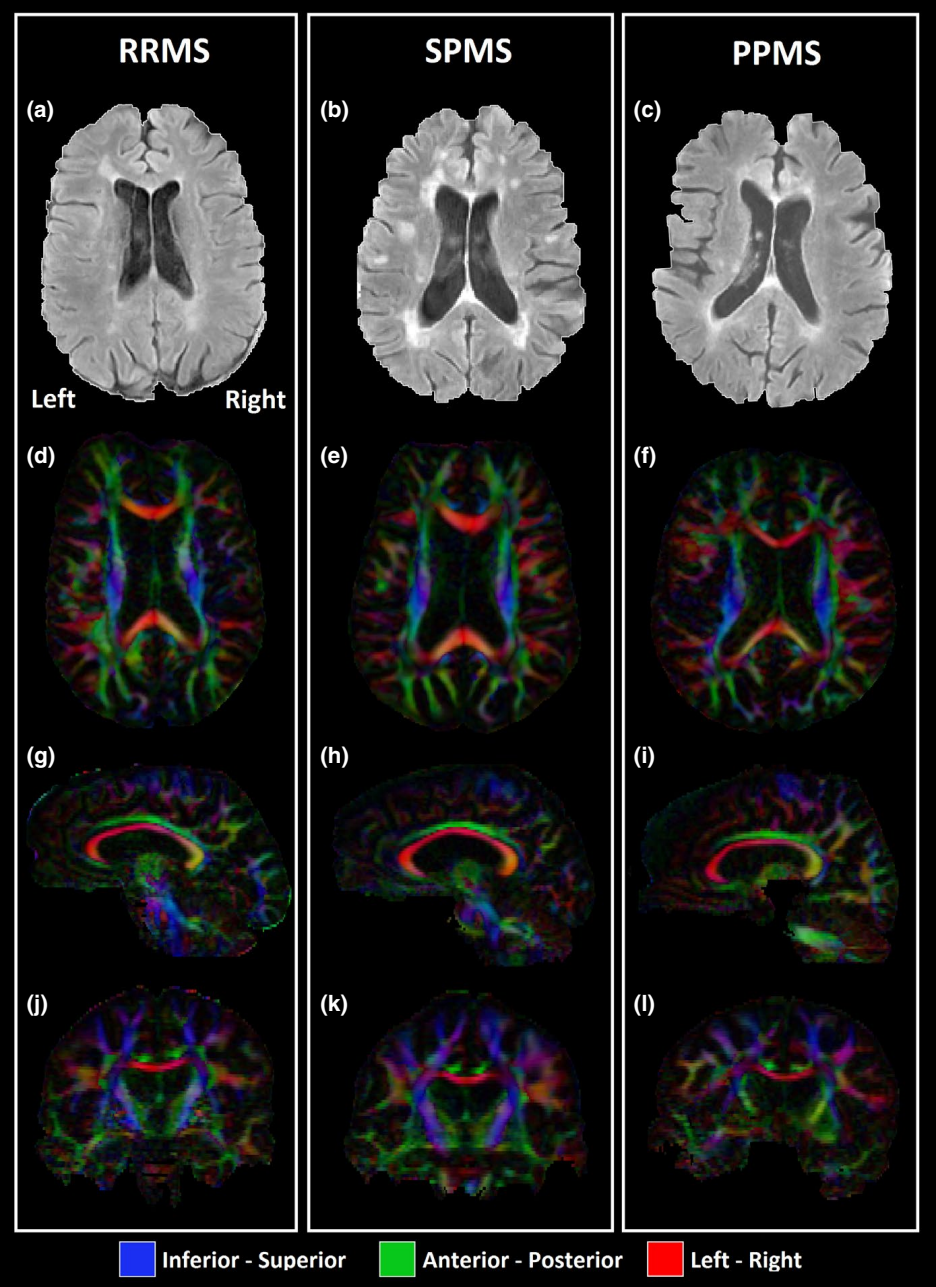
Axial FLAIR images showing periventricular lesions, and axial, sagittal, and coronal views of the 1.7 mm isotropic first eigenvector fractional anisotropy (FEFA) maps in a RRMS (a, d, g, j, 51 years old, male, lesion number = 24, total lesion volume = 8.8 cm^3^, time since diagnosis = 14 years, Expanded Disability Status Scale [EDSS] = 6.0), a SPMS (b, e, h, k, 52 years old, female, lesion number = 17, total lesion volume = 19.1 cm^3^, time since diagnosis = 24 years, EDSS = 6.5), and a PPMS patient (c, f, i, l, 54 years old, male, lesion number = 21, total lesion volume = 15.5 cm^3^, time since diagnosis = 3 years, EDSS = 6.5). Red—left/right, green—anterior/posterior, blue—inferior/superior

The control group and full MS cohort did not differ in sex (*p* = .74) and age (*p* = .38). There were differences between the three MS subgroups, namely RRMS were younger, had lower EDSS, and scored higher in PASAT, SPMS had longer time since diagnosis, and PPMS had more males than females and lower scores in PASAT. Lesion number and volumetric load were quite variable between MS participants and were not different between MS subgroups (Table 1), although SPMS and PPMS had some individuals with larger lesion volumes than the RRMS group. Lesion location patterns were similar across all MS subgroups (Figure 1a,b,c) mainly around and perpendicular to the body of the lateral ventricles and/or callosal junction areas (Dawson's fingers), consistent with the common lesion locations and shapes associated with the pathology. Within these groups, lesion numbers and volume did not correlate with age and sex although the lesion number showed a correlation trend toward statistical significance with age for the entire MS cohort (*r* = .36, *p* = .058). No significant associations were found between number of lesions and total lesion volumes with EDSS, time since diagnosis, or cognitive scores (Table 2).

**Table 2:** Partial Pearson correlation coefficients between right and left FA and volumes in the fornix (Fx), cingulum (Cg) and uncinate fasciculus (UF), total lesion volume (TLV), total lesion number (TLN), time since diagnosis, and clinical/cognitive scores over the entire MS cohort

Tract	Metric	Right/Left	Tract volume		TLV	TLN	Time since diagnosis	Clinical/cognitive tests				
			R	L				EDSS	BVMT‐R	MFIS	SDMT	BDI‐II
Fx	FA	R	**0.50 (0.0064)**	–	−0.44 (0.01)	**−0.57 (0.0015)**	−0.22 (0.25)	−0.06 (0.73)	0.18 (0.35)	−0.01 (0.92)	0.07 (0.73)	−0.24 (0.2)
		L	–	**0.61 (0.0004)**	**−0.49 (0.0068)**	−0.48 (0.0094)	−0.46 (0.01)	−0.08 (0.66)	0.08 (0.67)	−0.01 (0.94)	0.11 (0.57)	−0.27 (0.15)
	Vol	R	–	–	**−0.60 (0.0006)**	−0.48 (0.0095)	−0.17 (0.37)	0.02 (0.92)	0.36 (0.06)	0.08 (0.68)	0.25 (0.21)	−0.21 (0.27)
		L	–	–	**−0.59 (0.0007)**	**−0.51 (0.0055)**	−0.41 (0.02)	0.01 (0.92)	0.34 (0.07)	−0.10 (0.58)	0.27 (0.17)	−0.45 (0.01)
Cg	FA	R	0.26 (0.17)	–	−0.36 (0.06)	−0.48 (0.0095)	−0.18 (0.34)	0.30 (0.12)	0.39 (0.04)	−0.45 (0.01)	0.07 (0.72)	**−0.49 (0.0069)**
		L	–	0.46 (0.01)	−0.43 (0.01)	**−0.49 (0.008)**	−0.25 (0.19)	0.46 (0.01)	0.32 (0.09)	−0.26 (0.17)	0.03 (0.88)	−0.30 (0.11)
	Vol	R	–	–	−0.44 (0.01)	−0.42 (0.026)	−0.43 (0.01)	−0.04 (0.83)	0.23 (0.23)	0.03 (0.87)	−0.04 (0.83)	0.17 (0.37)
		L	–	–	**−0.55 (0.0019)**	−0.18 (0.35)	−0.18 (0.34)	−0.02 (0.9)	0.12 (0.51)	−0.04 (0.82)	−0.02 (0.92)	0.09 (0.63)
UF	FA	R	0.29 (0.12)	–	**−0.67 (0.0001)**	−0.45 (0.016)	−0.44 (0.01)	0.02 (0.92)	0.28 (0.14)	−0.04 (0.83)	0.21 (0.28)	−0.37 (0.04)
		L	–	**0.52 (0.0039)**	**−0.53 (0.0038)**	−0.21 (0.29)	−0.32 (0.09)	−0.06 (0.73)	0.18 (0.36)	0.11 (0.55)	−0.06 (0.75)	−0.15 (0.44)
	Vol	R	–	–	−0.41 (0.02)	−0.01 (0.94)	−0.28 (0.14)	−0.45 (0.02)	−0.10 (0.62)	0.24 (0.21)	0.07 (0.71)	−0.04 (0.82)
		L	–	–	**−0.58 (0.0012)**	−0.41 (0.032)	−0.33 (0.07)	−0.28 (0.16)	−0.13 (0.51)	0.44 (0.01)	0.17 (0.38)	0.00 (0.99)
–	TLV	–	–	–	–	0.39 (0.04)	0.41 (0.02)	0.26 (0.19)	−0.33 (0.08)	−0.10 (0.59)	−0.37 (0.05)	0.14 (0.46)
–	TLN	–	–	–	–	–	0.38 (0.047)	−0.01 (0.97)	−0.24 (0.22)	0.14 (0.48)	−0.24 (0.22)	0.26 (0.17)

Note

Correlation coefficients are controlled for age and sex in the 28 MS participants.

Bold values are false discovery rate (FDR)‐corrected (*p* ≤ .0086).

### 3.2 Qualitative assessment of the tracts

**Figure 2:**
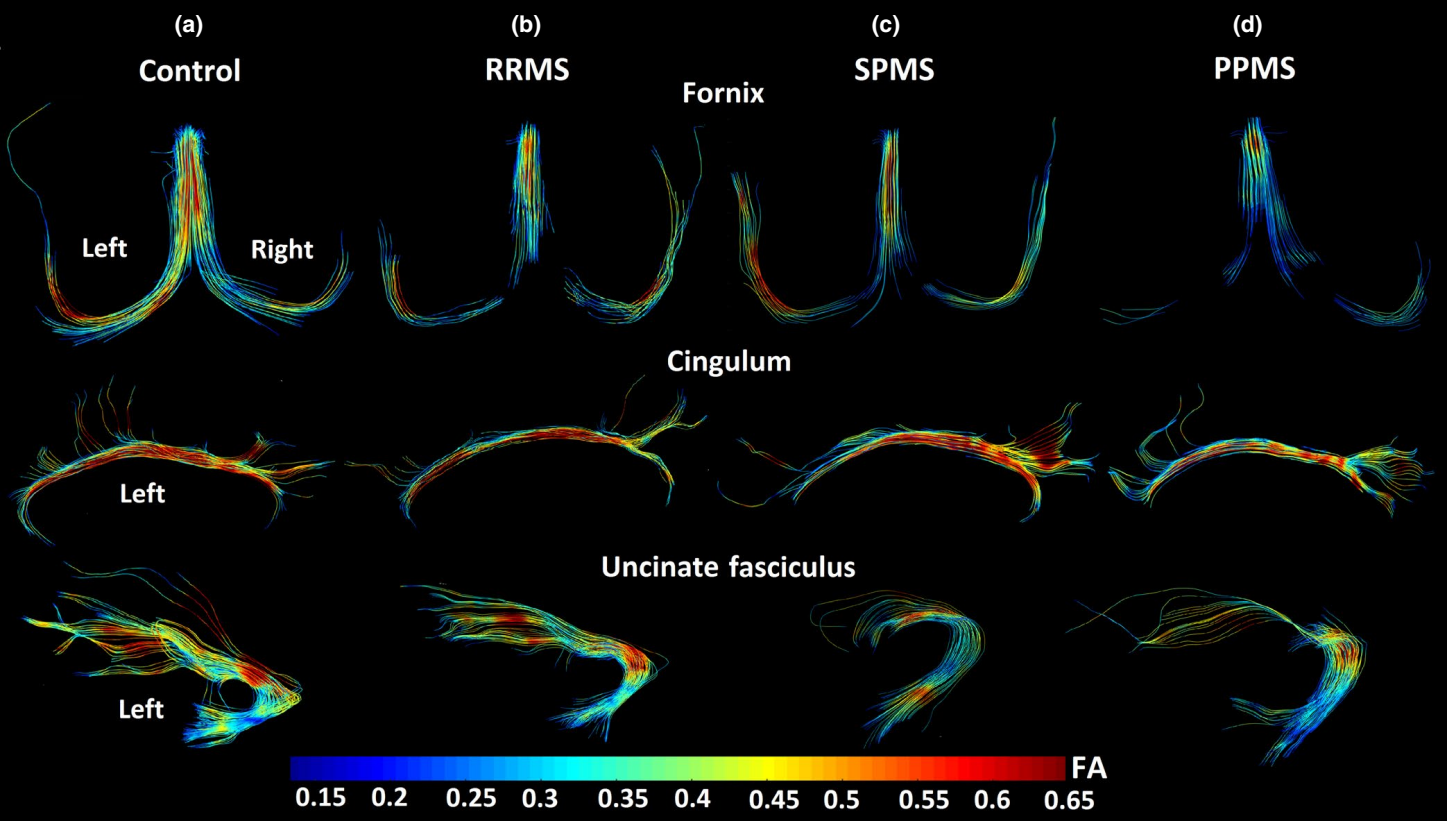
DTI tractography of the fornix (view from top), left cingulum, and left uncinate fasciculus (views from left side) with an FA color encoding scale for one example per group: (a) control, 50 years old, female; (b) RRMS, 48 years old, female, Expanded Disability Status Scale [EDSS] = 2.5; (c) SPMS, 59 years old, male, EDSS = 6.0; (d) PPMS, 54 years old, male, EDSS = 5.0. The fornix shows streamline disruptions and low FA in the three MS subtypes relative to controls. The cingulum tractography appears unaffected, while the uncinate fasciculus is quite variable in the extent of streamline projections to the anterior portion

When using the spatial resolution of 1.7 mm isotropic at high magnetic field of 4.7T (Figure 1d–l), the full extent of the fornix, with high FA, was achievable by DTI tractography in 10/11 healthy controls over ages of 21–62 years. The only exception was a 75‐year‐old female control whose fornix tractography was “transected” at the bilateral crus, but she is 9 years older than the oldest MS volunteer. In the case of the MS cohort, only 36% of the participants (3/11 in RRMS, 27%; 3/9 in SPMS, 33%; and 4/8 in PPMS, 50%) had the full extent of the fornix and in the remaining cases there were different degrees of tractography “transections” in all three MS subgroups (RRMS—1 left side, 2 right side, 5 bilateral; SPMS—2 right side, 4 bilateral; and PPMS—1 right side, 3 bilateral) with lower FA and fewer streamline projections (Figure 2b–d) relative to controls (Figure 2a). This current finding of incomplete fornix tracts is in spite of an ~40% voxel volume reduction compared to previous 8 mm^3^ voxel size typical in MS DTI studies at lower fields. These “transections”, even in one control, are caused by not reaching the FA threshold for tractography (0.2) due to partial volume with CSF leading to lower FA in the curvy and thin crus relative to the body (Figure 2b–d), when it passes through the lateral ventricles. These errors give partially reconstructed tracts and “artificial disconnections” that could be worse for MS if the fornix is thinner and smaller, increasing partial volume with adjacent CSF and stopping tracking.

In these same participants, the superior portion of the cingulum tracked well in all cases with no apparent qualitative differences in FA (Figure 2a–d). Tracking of the uncinate fasciculus was more variable (Figure 2a–d), and some MS participants showed shorter fibers than controls with fewer projections from the temporal lobe to the frontal lobe (4/28 MS patients—1 RRMS, left side; 2 SPMS, left side; and 1 PPMS, right side).

**Figure 3:**
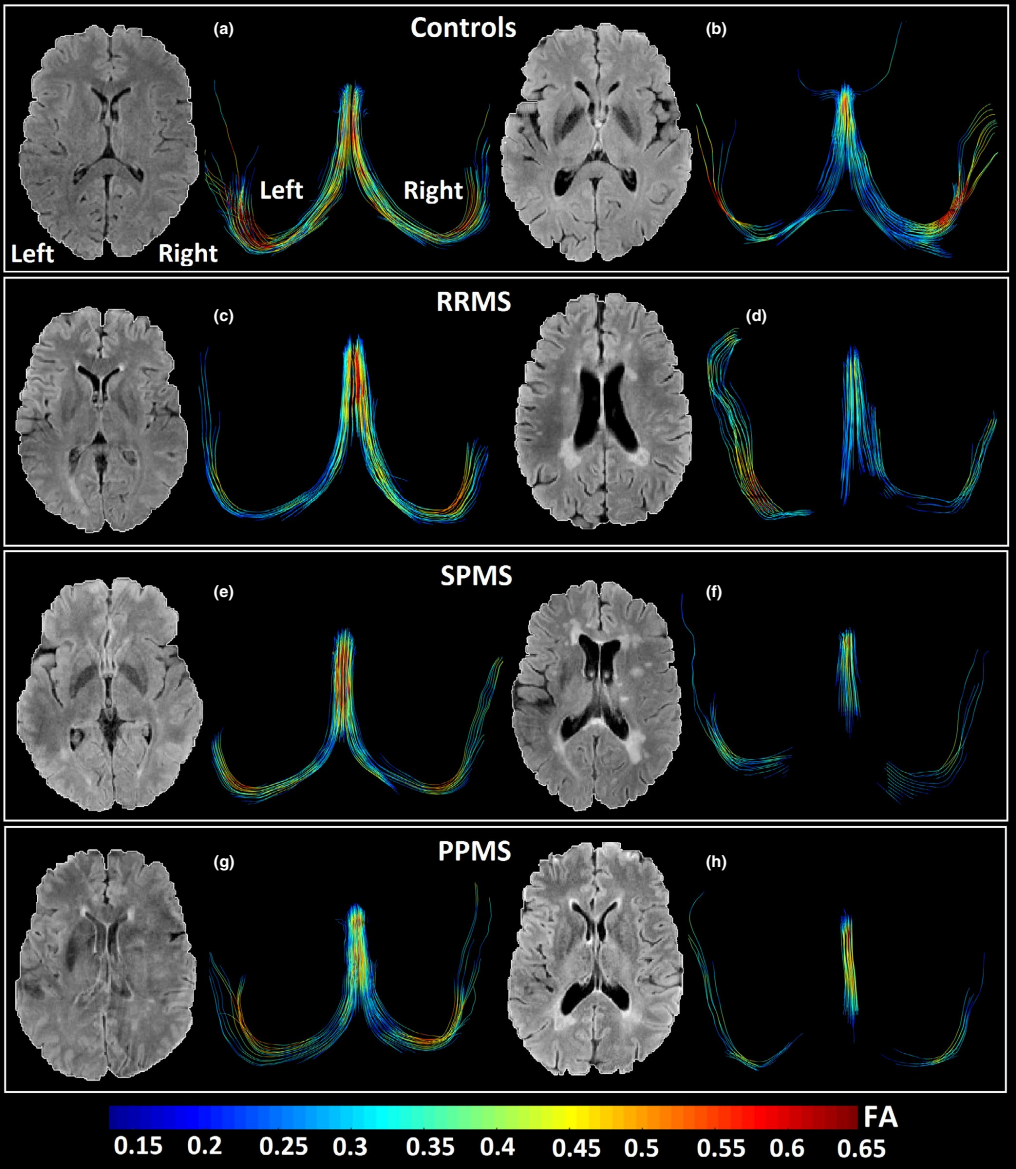
Two more examples of each subgroup including an axial FLAIR slice and the fornix for controls (a—21 years old, female; b—62 years old, female), RRMS (c—41 years old, female, EDSS = 2.5, time since diagnosis 1 year, TLV = 0.23 cm^3^; d—38 years old, female, EDSS = 1.5, time since diagnosis 10 years, TLV = 4.3 cm^3^), SPMS (e—45 years old, female, EDSS = 4.5, time since diagnosis 18 years, TLV = 0.19 cm^3^; f—66 years old, female, EDSS = 6.5, time since diagnosis 34 years, TLV = 27.9 cm^3^), and PPMS (g—65 years old, female, EDSS = 6.0, time since diagnosis 17 years, TLV = 0.83 cm^3^; h—59 years old, male, EDSS = 4.0, time since diagnosis 4 years, TLV = 7.5 cm^3^). MS participants with greater total lesion volume (f, h) have more disrupted fornix with lower volume and lower FA. The MS participants with a less disrupted fornix (c, e, g) have very low TLV (<1 cm^3^), and their age range and EDSS scores are quite variable

**Figure 4:**
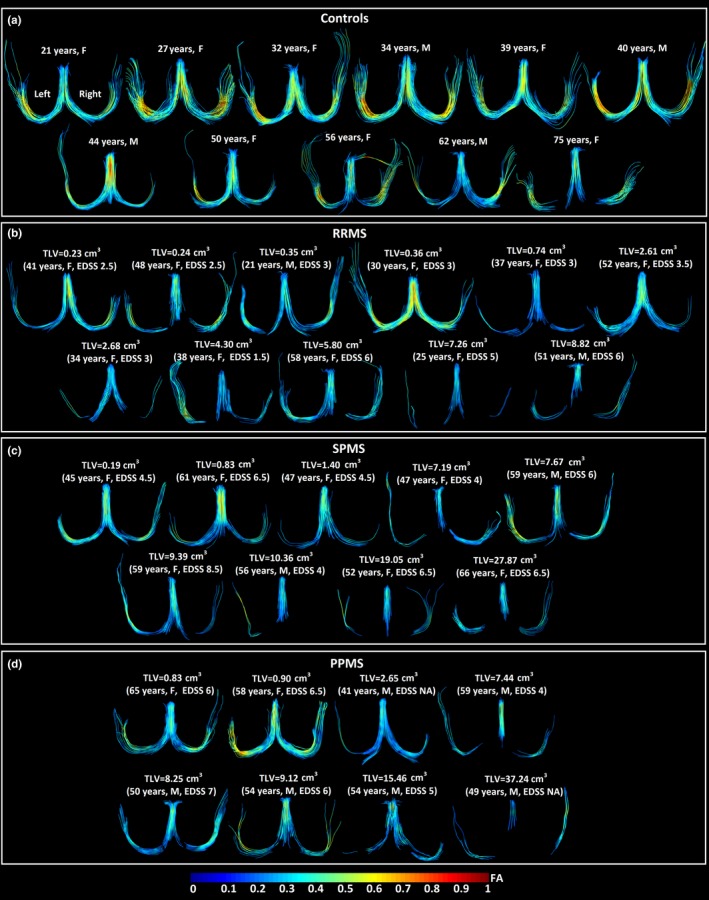
Tractography of the fornix for the entire cohort: (a) controls—ordered by age, (b) RRMS, (c) SPMS, (d) PPMS participants—ordered by total lesion volume (TLV) for each phenotype. The progressive nature of the fornix degradation and FA decrease is associated with global brain lesion progression in each of the RRMS, SPMS, and PPMS subgroups as shown by tractography here

Two more examples per group are shown to highlight nondisrupted (Figure 3a,b,c,e,g) and varying degrees of disrupted fornix (Figure 3d,f,h) on DTI tractography for the three MS subgroups. Transected fornices with apparently lower FA and volume appeared to be related with greater total lesion volumes and enlarged ventricles regardless of subgroup classification (Figure 3d,f,h, Figure 4b,c,d). Overall, for the entire MS cohort (Figure 4b,c,d), 4/13 participants with <3 cm^3^ total lesion volume (TLV) and 14/15 participants with more than 3 cm^3^ total lesion volume showed transected fornices. There is a progressive deterioration of the tracts in the MS cohort for all the phenotypes, which is coupled with FA decrease and it could be associated with greater total lesion volume in the whole brain. In the case of the controls, tracts are not deteriorated at the same degree but slightly lower volumes and FA could be qualitatively appreciated as the controls get older showing bilateral discontinuities in the oldest control due to aging (Figure 4a).

If the FA threshold for tracking is modified from 0.2 to 0.13, qualitative tract differences could be appreciated in the MS cohort primarily, due to a greater number of streamlines arising from the lower FA threshold. In some participants, this lower FA threshold enables streamlines to connect across the previously “transected” crura and there are fewer disconnections compared to the tracts previously presented here (FA threshold 0.13 vs. FA threshold 0.2): controls—no transections versus 1 bilateral transection; RRMS—4/11 transections (2 left side, 2 right side) versus 8/11 transections (1 left side, 2 right side, 5 bilateral); SPMS—5/9 transections (2 left side, 1 right side, 2 bilateral) versus 6/9 transections (2 right side, 4 bilateral); and PPMS—2/8 transections (1 left side, 1 bilateral) versus 4/8 transections (1 right side, 3 bilateral; Appendix: Figure S1).

### 3.3 Tract diffusion metrics and volumes

**Figure 5:**
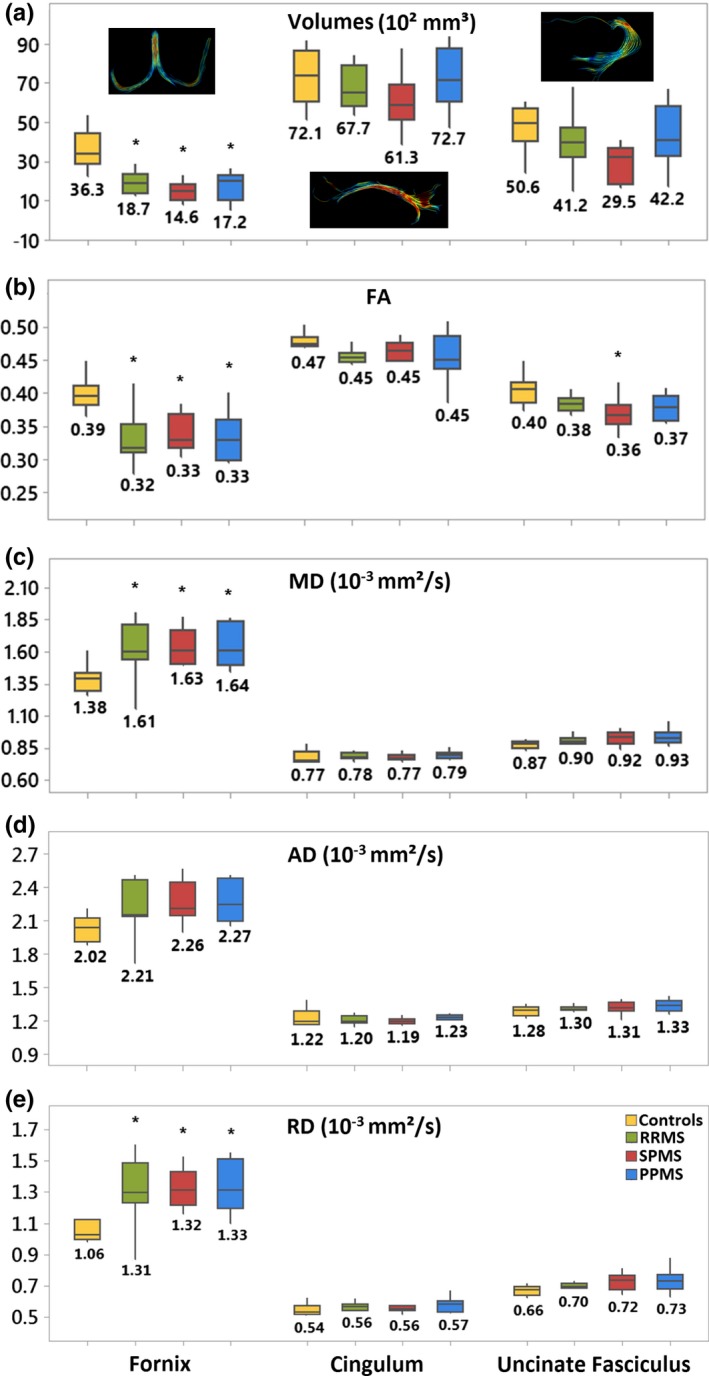
(a) Bilateral tract volumes, (b) FA, (c) MD, (d) AD, and (e) RD mean values are shown for controls, RRMS, SPMS, and PPMS subgroups for the fornix, cingulum, and uncinate fasciculus tracts. The central boxes show the median and interquartile range, while the whiskers above and below the boxes show the minimum and maximum values. The fornix shows significant differences between the controls and each MS subgroup for the volumes and all the diffusion metrics except AD. The cingulum did not show any differences. The uncinate fasciculus shows FA differences between the SPMS subgroup and the controls. *p ≤ .011 (FDR‐corrected for multiple comparisons)

**Figure 6:**
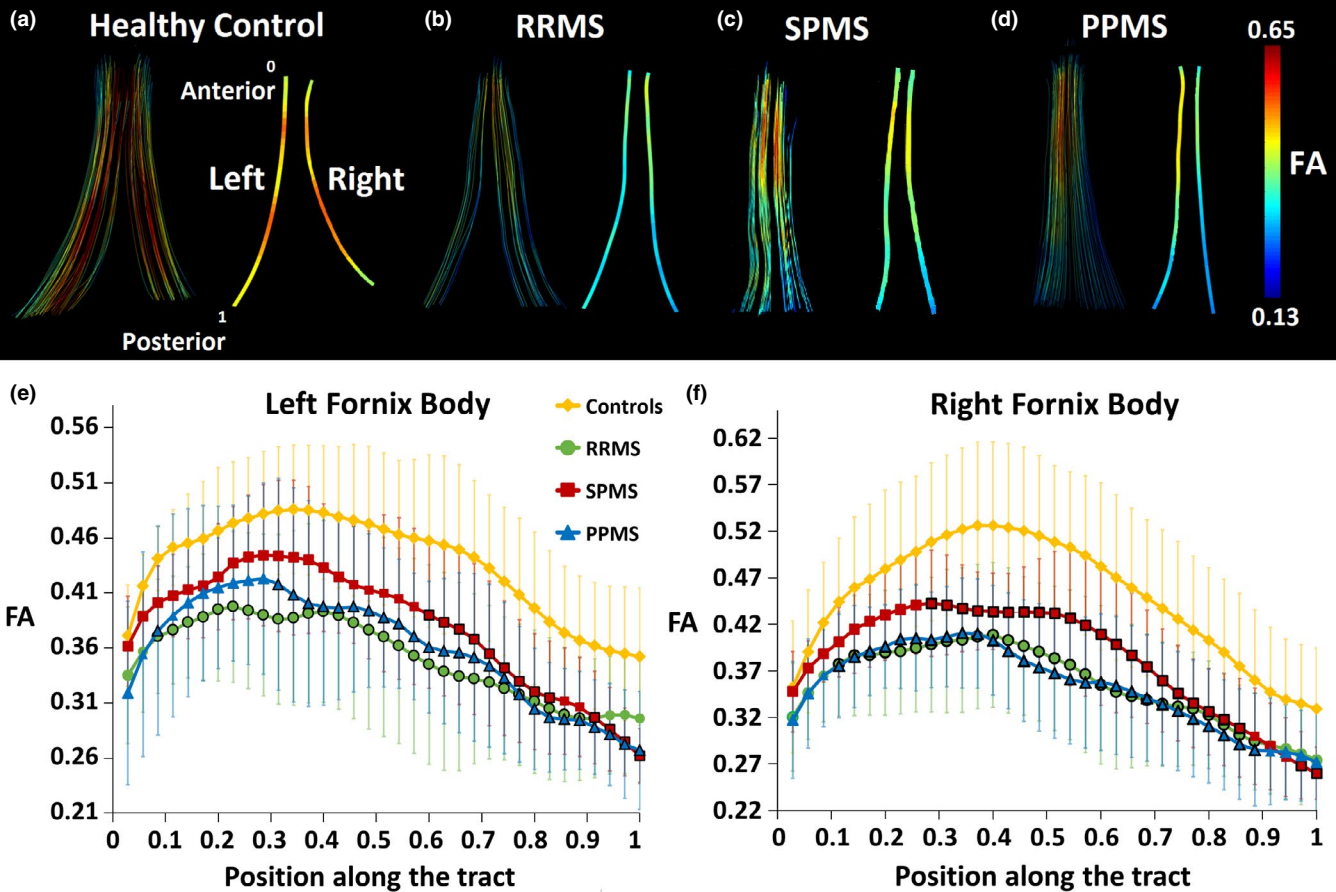
Between‐groups along‐the‐tract analysis in the left and right fornix body, a region which is identified in all 11 controls and 28 MS participants. The corresponding resampled‐averaged fornix body fibers are shown along 35 points (0 anterior to 1 posterior) in a healthy control (a, 40 years old, male), a RRMS (b, 41 years old, female), a SPMS (c, 47 years old, female), and a PPMS participant (d, 41 years old, male). (e, f) The along‐the‐tract FA profiles averaged over all participants in each group (mean ± *SD* shown) are significantly lower than the controls from 0.6 to 0.83 tract location area in the left fornix (e) and from 0.29 to 0.86 in the right fornix (f) for all three MS subgroups. All the statistically significant tract points are encircled with a dark black line for each MS subgroup compared to the controls (*p* ≤ .032 for the left fornix body, *p* ≤ .029 for the right fornix body, FDR‐corrected for multiple comparisons). Note that along‐the‐tract analysis was not possible in the bilateral crus of the fornix given so many “disconnections” in the tractography

The whole 28 MS cohort versus the 11 controls showed significant lower FA in the fornix (−16%, *p* < .001), the cingulum (−5%, *p* < .05), and the uncinate fasciculus (−7%, *p* = .001). The fornix and the uncinate fasciculus also showed smaller volume (−53%, *p* < .001; −25%, *p* = .023), greater MD (18%, *p* < .001; 5%, *p* = .006) and RD (24%, *p* < .001; 8%, *p* = .004), respectively, and higher AD (11%, *p* = .002) in the case of the fornix. Regarding the MS subgroups individually, the fornix showed significant group differences that were similar across all them, including smaller fornix volume (RRMS −49%, SPMS −60%, PPMS −53%), lower FA (RRMS −18%, SPMS −15%, PPMS −15%), greater MD (RRMS 17%, SPMS 18%, PPMS 19%), and higher RD (RRMS 24%, SPMS 25%, PPMS 26%) relative to controls (Figure 5). The cingulum did not show any differences in any of the three MS subgroups versus controls. The uncinate fasciculus showed significantly smaller FA (10%) in the SPMS group only. The along‐the‐tract analysis of the left and right fornix body, which is the superior region that is consistently tracked in all participants, showed significantly lower FA in the posterior portion of the left fornix and in the middle‐posterior portion of the right fornix for all three MS subgroups compared to controls (Figure 6).

Even if a lower FA threshold of 0.13 is applied in the entire healthy and MS cohort, which yields fewer “disconnections,” larger volumes, higher MD, and lower FA due to the inclusion of more voxels with low FA contributing to the tractography metrics, the main observations regarding group comparisons are similar to the previously reported comparisons for the FA = 0.2 threshold. The fornix volume/FA are still consistently abnormal in all three MS phenotypes compared with the controls, whereas MD/RD values were only significantly higher after the FDR correction for the SPMS subgroup (*p* ≤ .008; Appendix Figure S2).

### 3.4 *Fractional anisotropy and tract volumes *versus* clinical/cognitive scores*

**Figure 7:**
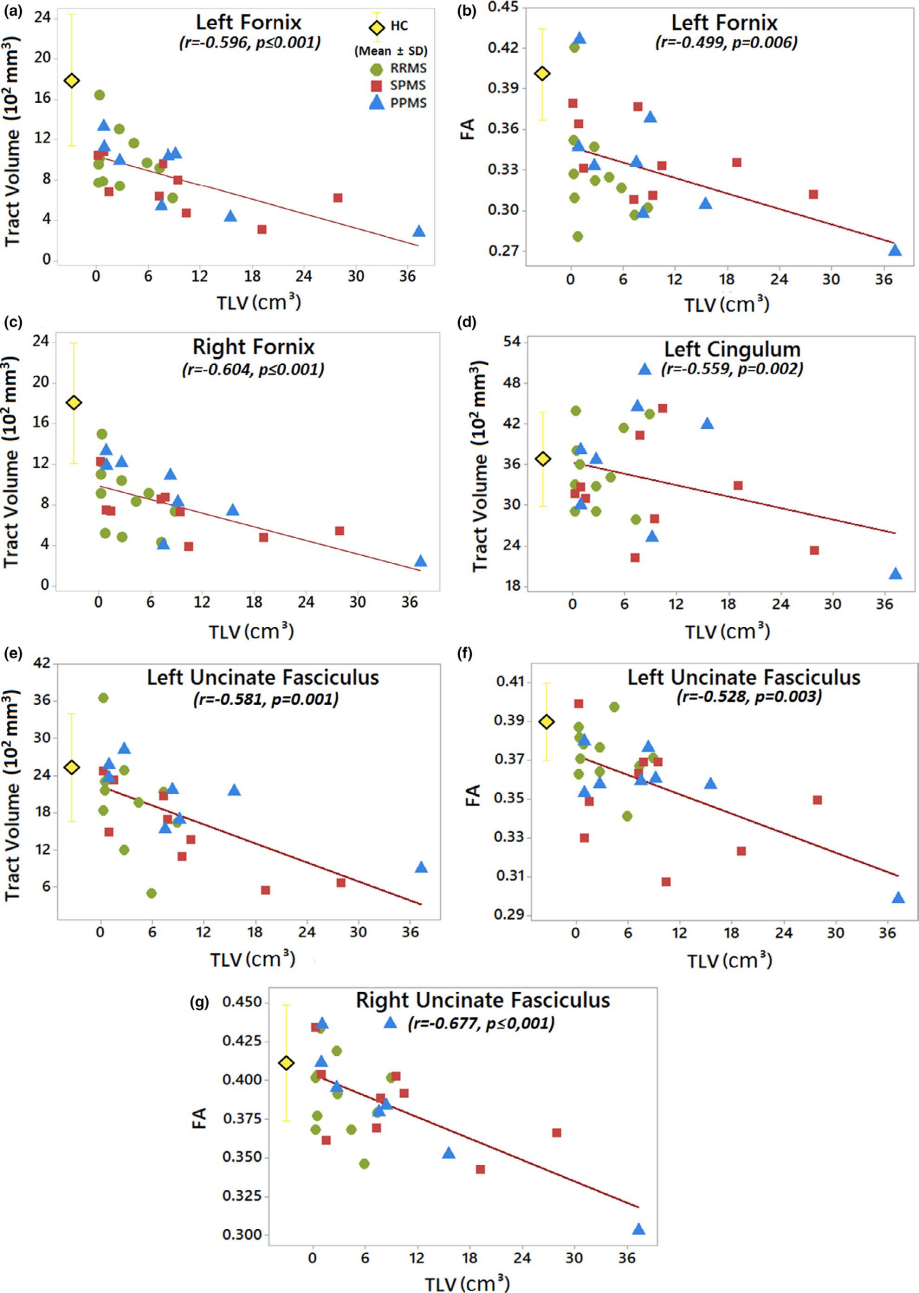
Greater total lesion volumes (TLVs) were correlated with smaller tract volumes for left/right fornix (a, c), left cingulum (d), and left uncinate fasciculus (e), as well as with lower FA values of the left fornix (b), and left/right uncinate fasciculus (f, g) (*p* ≤ .0086) for all three subgroups of MS (color legend) combined. The mean and the standard deviation of the healthy controls (HC) group are shown as reference

**Figure 8:**
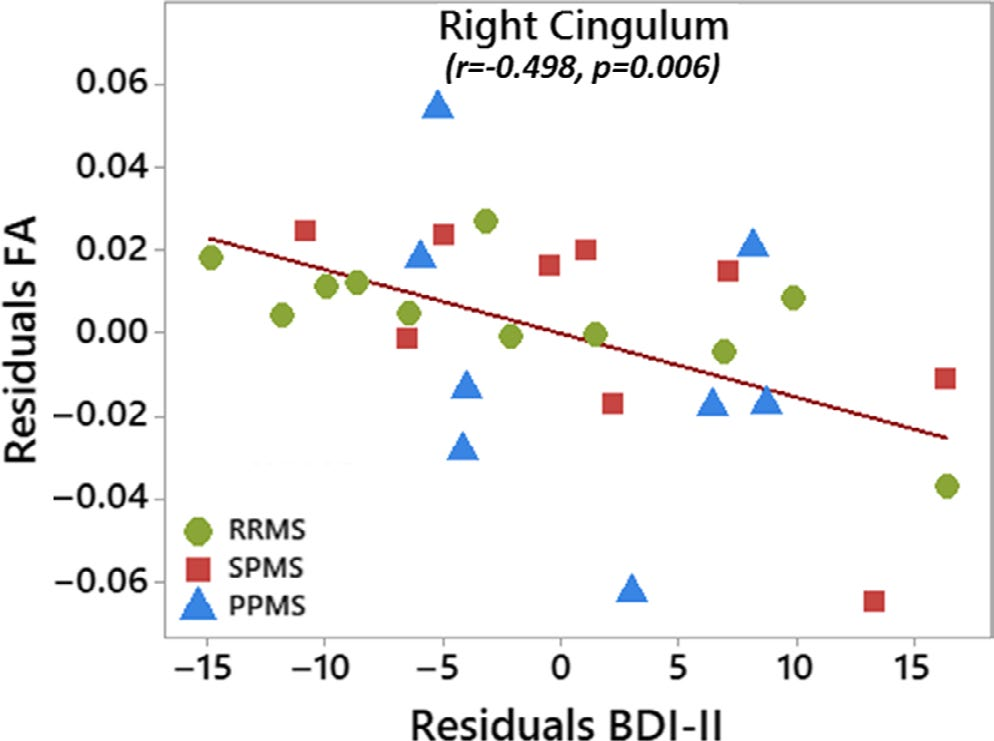
Lower FA of the right cingulum correlated with more depression (i.e., higher BDI‐II) scores, both corrected for age and sex, over all 28 MS participants

FA correlated positively with tract volume for the left and right fornix as well as the left uncinate fasciculus (Table 2). Notably, total lesion volume throughout the brain correlated negatively with several measures of FA and tract volume primarily for the fornix and uncinate fasciculus, namely left FA and left/right volume of the fornix, left volume of the cingulum, and left/right FA and left volume of the uncinate fasciculus (Figure 7, Table 2). There are four participants (two SPMS and two PPMS) with total lesion volumes each >15 cm^3^ that appear to be driving the negative correlations. Lower FA values in the right cingulum correlated with more severe depression in the entire MS cohort (Figure 8, Table 2). There are seven participants (one RRMS, three SPMS, and three PPMS) that appear to be driving the correlations with depression. They all similarly showed high fatigue scores and five of them (two SPMS and three PPMS) also showed lesion volumes >7 cm^3^.

## 4 DISCUSSION

In agreement with previous MS DTI studies primarily focused on RRMS that have implicated fornix injury (Dineen et al., 2012, 2009; Fink et al., 2010; Kern et al., 2012; Keser et al., 2017; Roosendaal et al., 2009; Syc et al., 2013; Yu et al., 2012), tractography of the fornix acquired with 1.7 mm isotropic resolution showed markedly lower volume (by ~54%) and FA (by ~16%), as well as higher MD (by ~18%) and RD (by ~25%) to a similar extent in RRMS, SPMS, and PPMS relative to controls. The fornix DTI abnormalities were present in all three MS cohorts despite the disparate ranges of 21–66 years of age, 1–34 years since diagnosis, or 1.5–8.5 EDSS, implying its widespread occurrence early on in the disease course. The three following DTI studies on SPMS and/or PPMS have implicated the fornix, but this tract was not their focus nor was its 3D trajectory identified and measured using tractography (Bodini et al., 2009; Meijer et al., 2016; Preziosa et al., 2011). Whole‐brain TBSS analysis yielded lower FA and higher RD of the fornix in SPMS with and without cognitive impairment, with the former showing greater changes (Meijer et al., 2016). Fornix FA was reduced in PPMS, but data were not specific for this region (Bodini et al., 2009). One of the few studies to report DTI changes in all three MS diagnostic subpopulations published voxel‐based statistical significance figures that suggest fornix anomalies in SPMS and PPMS as well, and the fornix appeared to have reduced FA in PPMS versus healthy controls and in SPMS versus benign MS (Preziosa et al., 2011).

It is unclear why all three MS subtypes would yield similar changes to the fornix. Histopathologic studies have reported comparable axon damage across the different subtypes, such as in the corticospinal tract in SPMS and PPMS (Tallantyre et al., 2009) or in demyelinating and active lesions (Kuhlmann, Lingfeld, Bitsch, Schuchardt, & Brück, 2002). The clinical presentation does not always allow an accurate differentiation between relapsing and progressive phases in the disease spectrum (Kuhlmann, 2013). Over the entire MS group, however, the left/right fornix volume and left fornix FA were negatively correlated with total lesion volume suggesting a link between the degree of visible whole‐brain lesion pathology and fornix integrity. Periventricular lesions are common in MS and are suggestive that inflammatory factors in the CSF may be involved (Matejčíková et al., 2015). The fornix would be greatly exposed to such factors since it is bathed in CSF as it passes through the lateral ventricles which could explain the abnormal fornix tractography findings even in those with brief time since MS diagnosis. White matter fornix FA has been linked to Alzheimer's disease‐related CSF factors in normal adults (Gold et al., 2014). Active and chronic inactive lesions of the fornix have been observed on histology in 9/16 and 7/16 mostly RRMS participants, respectively (Huitinga et al., 2001). One MRI study only reported 2.6% of 156 RRMS participants with fornix lesions on FLAIR (Sahin, Selouan, Markowitz, Melhem, & Bilello, 2016), but it was limited by low resolution of the coregistered FA color maps used to confirm lesion location and potential linear registration algorithm errors. Demyelination of the hippocampus has been reported in brain tissue histological sections of 12/22 progressive MS participants (nine SPMS, three PPMS; Dutta et al., 2011) and this could be reflected in Wallerian degeneration to axons in its major primary efferent fiber bundle, the fornix, although the relationship is still unclear and needs further investigation. The fornix diffusion MRI metrics did not correlate with any of the clinical or cognitive measures, unlike earlier studies (Dineen et al., 2012, 2009; Fink et al., 2010; Kern et al., 2012; Koenig et al., 2013; Syc et al., 2013; Yu et al., 2012).

Tract‐based spatial statistics (TBSS) mentioned above in many MS DTI studies is a commonly used voxel‐based analysis method that involves skeletonization of the white matter and projection of maximal FA values nearby to the skeleton, but it has been shown to have difficulties in the assessment of a small, curvy tract such as the fornix (Bach et al., 2014). Tractography has its advantages since it is performed in native space which allows anatomical variations in shape and location, and does not require spatial normalization of the images to a common template. However, a limitation of tractography is that it can lead to discontinuities of tract streamlines when the voxel FA or eigenvector angle thresholds are not met, thereby not identifying voxels that are the most severely affected. For the fornix, which is an isolated bundle without other crossing fibers, this tracking stoppage usually occurs when a voxel falls below the set threshold due to partial volume effects with isotropic CSF in the bilateral crus and leads to these apparent discontinuities even in healthy controls (Concha et al., 2005). None of the previous fornix DTI papers of MS used any CSF suppression methods—acquisition or postprocessing. Furthermore, the implementation of FLAIR‐DTI was not possible at 4.7T and the single shell DTI data used here are not suitable for postprocessing “free water elimination (FWE)” CSF correction algorithms that require two shells (Hoy, Koay, Kecskemeti, & Alexander, 2014). These FWE algorithms are not still the optimal approach, compared to FLAIR‐DTI, to further reduce the standard deviation of diffusion metrics for tracts with high partial volume effects with CSF, such as the fornix (Hoy, Kecskemeti, & Alexander, 2015).

Certainly, fornix tractography is susceptible to stopping its tracking for the above‐mentioned reasons but this is not necessarily detrimental. Even if one assumes that the fewer tracts are just due to CSF contamination, then this would imply smaller actual fornix that would increase CSF partial volume and stop tracking. If the fornix volumes were the same, then the error ought to remain consistent in MS as in controls; but it is not the same. Thus, there will be a link between the actual volume and that which is underestimated with tractography. The lower tracking FA threshold of 0.13 yields larger fornix tracts, smaller FA, and higher MD/AD/RD for all four subgroups (controls, RRMS, SPMS, and PPMS). The group differences of the fornix hold for all three MS subgroups relative to controls for FA and volume with this lower FA threshold, but only the SPMS group remains significantly different for MD and RD relative to controls. Since the same DTI acquisition and postprocessing algorithms with the exact same tracking ROIs and thresholds were applied in all MS participants and controls, these discontinuities indicate markedly reduced FA in these areas of the fornix within the MS patients and any tractography differences are still indicative of group differences of the underlying fornix. This could be due to either actual microstructural differences of the residual fornix in the crus, small demyelinating lesions going undetected due to low FLAIR resolution, Wallerian degeneration from potentially damaged hippocampal areas, or simply greater partial volume effect with adjacent CSF if the fornix has undergone greater atrophy (“normal” microstructure but smaller volume). Table 2 shows a positive correlation of left and right fornix FA with tractography‐derived fornix volume. Studies are currently underway here to evaluate the fornix in MS using FLAIR‐DTI to suppress the CSF, minimize partial volume effects, and yield more complete tracking of the fornix even in the case of atrophy (Concha et al., 2005). However, the “along the tracts” analysis of the consistently tracked superior body of the fornix in all controls and MS participants suggests that there are microstructural fornix differences, since the body is not as susceptible as the crus to partial volume errors with CSF.

The uncinate fasciculus had lower FA only for SPMS when compared to controls, consistent with a previous study focused on that phenotype (Meijer et al., 2016). Others have reported lower FA of the uncinate fasciculus in RRMS (Kern et al., 2015; Pardini, Bergamino, et al., 2014; Yu et al., 2012) or mixed MS cohorts (Fink et al., 2010; Keser et al., 2017; Mesaros et al., 2012; Preziosa et al., 2011). The uncinate fasciculus tractography was quite variable in our MS participants; for example, in one RRMS, two SPMS, and one PPMS participant, it did not show frontal lobe projections, unlike all the healthy controls. These variations may be related either to lesions in frontal cortex areas that this tract connects (Bodini et al., 2009) or to other parts of its pathway that could affect the tracking. The uncinate fasciculus volume and FA also showed significant correlations with total lesion volume.

The cingulum did not show any group diffusion differences relative to controls in agreement with prior work (Kern et al., 2012; Roosendaal et al., 2009) although several other MS studies have reported cingulum differences, mainly FA (Dineen et al., 2009; Kern et al., 2015; Keser et al., 2017; Mesaros et al., 2012; Preziosa et al., 2011; Syc et al., 2013; van Geest et al., 2018; Yu et al., 2012). In our study, lower FA in the right cingulum correlated with higher depression scores, possibly due to interactions between the anterior cingulate and the amygdala (Bubb, Metzler‐Baddeley, & Aggleton, 2018). This finding is consistent with lower cingulum FA in young women at risk of depression (Keedwell et al., 2012), and lower FA in the right parahippocampal cingulum in those with catechol‐O‐methyltransferase (COMT) gene polymorphisms in major depressive disorder (Seok et al., 2013). Lower FA in cingulum has correlated with deficits of episodic and working memory (Dineen et al., 2009; Mesaros et al., 2012; Syc et al., 2013; Yu et al., 2012) and subjective fatigue (Pardini, Bonzano, et al., 2014) in MS, but these studies did not measure depression. A TBSS study showed lower FA of the left cingulum in depressed MS patients (van Geest et al., 2018) and DTI metrics in NAWM have been related to depression in MS (Feinstein et al., 2010).

The main technical limitation of our study is the lack of a CSF‐suppressed DTI sequence; therefore, the fornix tractography may have been altered by partial volume effects from CSF in the ventricles making the DTI metrics and tractography prone to errors and harder to interpret. The authors are currently utilizing high‐resolution FLAIR‐DTI at lower magnetic field strength (3T) in new MS cohorts in order to diminish the CSF confounding effects in the DTI metrics. While FLAIR‐DTI works fine at these lower magnetic field strengths, there are specific absorption rate (SAR) constraints at higher fields such as 4.7 T used here which have resulted in the development of alternative strategies to minimize the influence of CSF in DTI (Baron & Beaulieu, 2015), but this was not used in the present study. Furthermore, the small sample size of this study, particularly when looking at each RRMS, SPMS, and PPMS subgroup separately, is another limitation to consider when making inferences about differences between these clinical groups and it should be addressed in future studies. The heterogeneity of the MS subgroups, the high participant variability of the cognitive, depression and fatigue test scores, and the burden of cognitive impairment altogether with the small sample size of the MS cohort may have contributed to the inability to find more cognitive and clinical associations with the DTI findings.

## 5 CONCLUSIONS

In summary, high‐resolution diffusion MRI tractography identified abnormalities in the fornix that were similar across all three primary MS phenotypes of RRMS, SPMS, and PPMS regardless of EDSS, time since diagnosis, or cognitive scores. The fornix FA and tract volumes were more affected with greater total whole‐brain lesion volumes suggesting a link to other brain pathology that needs further investigation.

## CONFLICT OF INTEREST

The authors have no conflict of interest to declare.

## Data Availability

The data that support the findings of this study are available from the corresponding author upon reasonable request.
