# Supplementary figures and images for: Diffusion tensor imaging tractography reveals altered fornix in all diagnostic subtypes of multiple sclerosis
Source: Brain Behav. 2019 Dec 19;10(1):e01514. doi: 10.1002/brb3.1514 (PMC6955822; doi:10.1002/brb3.1514)

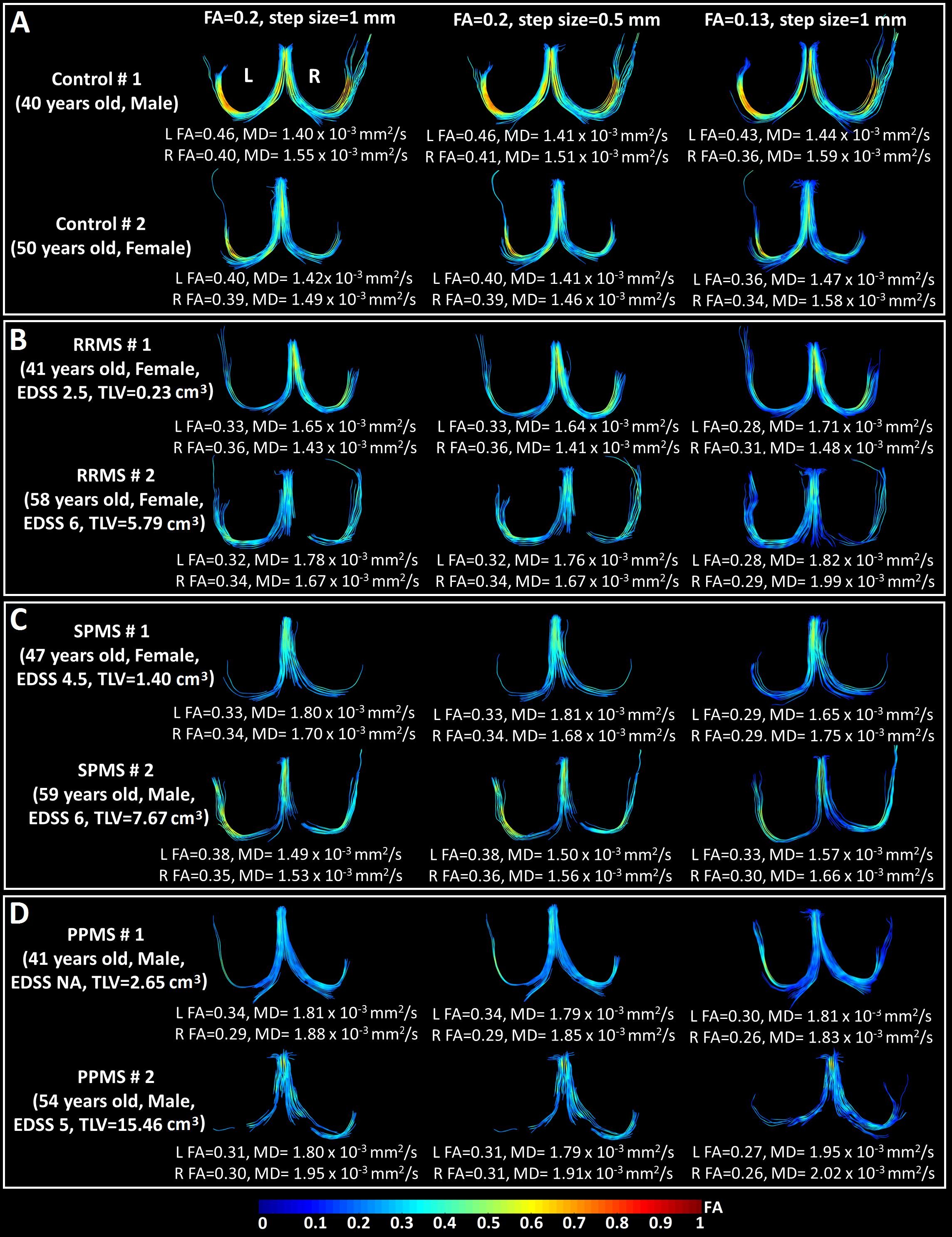

Supplement: Supplementary file 1 [file BRB3-10-e01514-s001.tif]

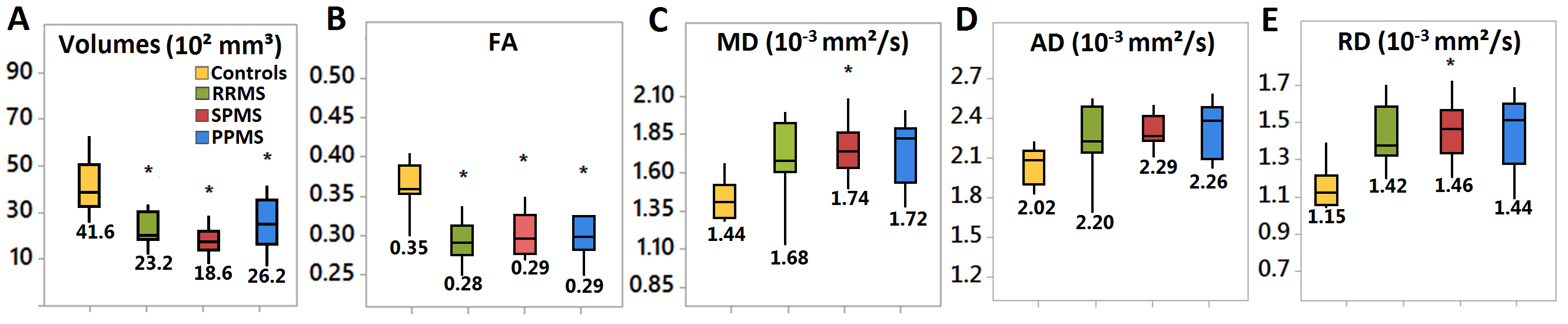

Supplement: Supplementary file 2 [file BRB3-10-e01514-s002.tif]
